# Effects of Texture Modifiers on Physicochemical Properties of 3D-Printed Meat Mimics from Pea Protein Isolate-Alginate Gel Mixture

**DOI:** 10.3390/foods11243947

**Published:** 2022-12-07

**Authors:** Supanut Leelapunnawut, Luxsika Ngamwonglumlert, Sakamon Devahastin, Antonio Derossi, Rossella Caporizzi, Naphaporn Chiewchan

**Affiliations:** 1 Advanced Food Processing Research Laboratory, Department of Food Engineering, Faculty of Engineering, King Mongkut’s University of Technology Thonburi, 126 Pracha u-tid Road, Tungkru, Bangkok 10140, Thailand; 2 The Academy of Science, The Royal Society of Thailand, Dusit, Bangkok 10300, Thailand; 3 Department of Agriculture, Food Natural Resources and Engineering, University of Foggia, Via A. Gramsci 89/91, Via Napoli 25, 71122 Foggia, Italy

**Keywords:** animal meat, cooking loss, κ-carrageenan, rheological properties, textural properties, transglutaminase

## Abstract

Meat mimics were prepared from pea protein isolate-alginate gel via 3D printing. The texture of 3D-printed meat mimics was modified by incorporating transglutaminase (TGase) or κ-carrageenan (κc) at 0.3, 0.6 or 0.9% (*w/w*) into printing material prior to 3D printing. Rheological properties of modified printing material were measured; results were used to support 3D printing results. Textural properties of raw and cooked meat mimics were determined and compared with those of selected animal meats, namely, pork tenderloin, chicken breast, salmon meat and Spanish mackerel. Cooking losses of meat mimics were also determined. *G′*, *G″* and tan δ of TGase-modified material were not significantly different from those of the control. In contrast, increasing κc content resulted in increased *G′* and *G″*; tan δ of all κc-modified samples decreased from that of the control. Addition of TGase at 0.9% into printing material increased the hardness of raw meat mimics, while κc at 0.9% increased hardness of cooked meat mimics. Raw meat mimics treated with 0.9% TGase exhibited texture closest to raw salmon. Texture of cooked meat mimics treated with 0.9% κc was closest to that of cooked salmon. TGase-treated meat mimics tended to experience lower cooking losses, while κc-treated meat mimics exhibited an opposite trend.

## 1. Introduction

Over the past decade, the production and consumption of animal meat and meat products have continuously been on the rise due to the rapid growth of the human population [1]. Since natural resources have limited abilities to respond to such an increasing demand, the situation is clearly unsustainable. Moreover, animal farming is known to adversely affect the environment, contributing in a significant way to greenhouse gas emissions and climate change; ethical issues related to farming and slaughtering have also emerged as other important concerns [2]. An alternative to animal meat is therefore desirable. Development of plant-based meat mimics is one possible option that may help reduce animal meat consumption. Nevertheless, although plant-based meat mimics are now available, the major challenge facing plant-based meat mimics is their ability to obtain acceptance from most consumers [2,3]. One of the most important concerns related to the acceptance of plant-based meat mimics is their texture, which is clearly related to how meat mimics are formulated and formed [4,5]. A number of techniques can be used to produce meat mimics, including wet spinning, electrospinning, thermal extrusion, freezing structuring and shear cell technology. Extrusion, however, exhibits high commercial feasibility and consists of several sub-techniques that allow the production of fibrous structure imitating animal meat structure [6]. Among extrusion-based techniques, extrusion-based 3D printing is one of the most promising for the production of plant-based meat mimics.

To prepare plant-based meat, protein isolated from soy [7,8,9], maize [10] or pea [11] is commonly used. Pea protein isolate is of particular interest as it is less allergenic [12]. Pea protein isolate also contains a well-balanced amino acid profile [13]. Suitable addition of pea protein isolate has indeed proven to improve the nutritional balance of many food products. Besides plant protein isolate, hydrocolloids are another important ingredient typically required to improve printability of a 3D printing material as well as shape retention of a 3D-printed product [14]. Among the many available types of hydrocolloids, sodium alginate is widely used because of its high viscosity and superior gelling property [15]. The addition of sodium alginate helps to maintain the shape/structure of a 3D-printed product through ionic crosslinking with a divalent or trivalent ion, e.g., calcium ion [16,17].

Although a mixture of pea protein isolate and alginate gel exhibits self-support potential after 3D printing, its printed texture is still dissimilar to that of animal meat [18]. To better imitate animal meat, a texture modifier should be added. Methylcellulose, e.g., carboxymethylcellulose, is one of the most commonly used texture modifiers. However, there have recently been some concerns about its negative effects on gut microbiota [19]. Other types of texture modifiers, including enzymes, are therefore of interest. Transglutaminase (TGase) is an interesting candidate enzyme as it can induce bond formation between protein molecules [20]. TGase has already been used to modify the texture of an array of plant-based meat mimics. Setiadi et al. [21], for example, investigated the effect of TGase addition on the characteristics of meat mimics prepared from vegetable protein. The use of TGase successfully helped to modify the texture of the samples in terms of hardness, cohesiveness and springiness. Besides enzymes, algae-derived hydrocolloids, e.g., κ-carrageenan (κc), can also be used to modify the texture of plant-based meat mimics; κc allows adhesion of matrix materials to form a more homogeneous structure [20]. The use of κc to improve the texture of plant-based patties was, for instance, investigated by Tunnarut et al. [22]. κc was noted to be capable of improving the texture of the product in terms of hardness and chewiness. However, limited studies, if any, are available on the use of texture modifiers in combination with 3D printing to prepare plant-based meat mimics.

The present study aimed to prepare plant-based meat mimics from a mixture of pea protein isolate and alginate gel via the use of 3D printing. The effects of selected texture modifiers, i.e., TGase and κc, at different concentrations on textural properties (in terms of hardness, chewiness, springiness, adhesiveness and cohesiveness) of 3D-printed meat mimics were evaluated. Textural properties of both raw (uncooked) and cooked meat mimics were compared to those of some raw and cooked animal meats, i.e., pork tenderloin, chicken breast, salmon and Spanish mackerel. Cooking losses of pan-fried meat mimics of various formulations at 150 °C for 10 min were also determined.

## 2. Materials and Methods

### 2.1. Materials

Commercial pea protein isolate powder, which contains 82% protein, 11% carbohydrate and 0.5% fat, was purchased from www.clubproteinonline.com. Alginate powder, sodium phosphate and calcium chloride were purchased from Chemipan (Bangkok, Thailand). TGase was obtained from Ajinomoto (Tokyo, Japan), while κc was purchased from Krungthepchemi (Bangkok, Thailand).

### 2.2. Preparation of Printing Material

Preparation of the printing material was conducted as per the modified methods of Oyinloye and Yoon [11]. Calcium alginate gel was first prepared prior to being mixed with pea protein isolate. Gel preparation started by mixing 1.5 g of sodium alginate with 0.25 g of sodium phosphate and 98 g of distilled water; the contents were stirred at 43 °C for 40 min. The mixture was then added to 0.25 g of calcium chloride and introduced to a high-shear homogenizer (IKA, T-25 Ultra Turrax, Staufen, Germany) at 15,000 rpm for 1 min. Note that alginate was used because of its gelation and reversible modulus, which are suitable for 3D printing. Calcium chloride was added to increase the gel strength, while sodium phosphate was added to control the rate of gelation.

In the preliminary experiments, which were conducted to determine the optimal ratio of alginate to pea protein isolate, alginate gel was mixed with pea protein isolate at different mass ratios, i.e., 100:20, 100:25 and 100:30, using a mixer (KitchenAid, K5SS, Greenville, OH, USA) for 3 min. Each obtained paste was kept in a beaker and placed in a refrigerator at 4 °C for 24 h. Prior to 3D printing, the paste was placed in a water bath at 25 °C for 1 h. Based on visual observation, the paste prepared from alginate gel and pea protein isolate at a ratio of 100:25 was most suitable for 3D printing; the printed structure could adequately maintain its shape and did not collapse. This composition was therefore selected as the printing material formulation in the subsequent experiments.

### 2.3. Modification of Printing Material

In an attempt to imitate the texture of animal meat, the aforementioned printing material was modified. Texture modifier, namely, TGase or κc, was manually mixed into the printing material at a concentration of 0.3, 0.6 or 0.9% (*w/w*) prior to being measured for its rheological properties. No incubation or heat treatment was performed at this stage. The control sample was the printing material without TGase or κc.

### 2.4. Rheological Properties Measurement of Printing Material

Rheological properties measurement of the printing material was conducted by using a rheometer (Thermo Fisher Scientific, HAAKE MARS III, Karlsruhe, Germany) with a cone-and-plate geometry (cone diameter of 35 mm and cone angle of 2°). The printing material was placed in a 0.105-mm gap between the cone and plate at 25 °C for 5 min prior to each measurement to relax the residual stresses of the material. The perimeter was covered with paraffin oil to prevent dehydration of the material during the measurement. Small amplitude oscillatory strain sweep (0.001–100%) at a frequency of 0.1 Hz was conducted to determine the linear viscoelastic region at a constant temperature of 25 °C. The strain noted at 0.01% deformation was used to evaluate the storage modulus (*G′*) and loss modulus (*G″*) of the material using the frequency sweep test within the range of 0.01–10 Hz at 25 °C.

### 2.5. 3D Printing

**Figure 1:**
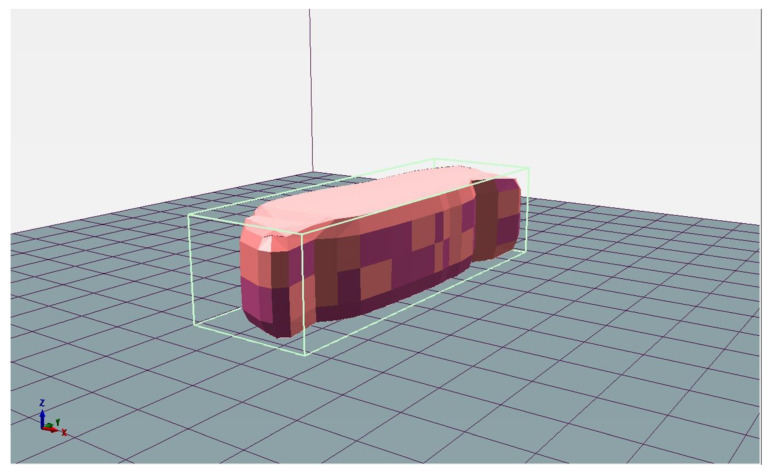
3D model of meat mimics.

A 3D model of a steak cut (Figure 1), which was obtained from www.turbosquid.com, was used as the digital model for printing meat mimics with the dimensions of 20 × 80 × 25.4 mm. Printing was conducted using a Foodbot D2 3D printer (Shiyin, Hangzhou, China). The printer was controlled by a personal computer installed with Repetier Host 2.1.2 software (Hot-World GmbH and Co. KG, Willich, Germany).

In order to obtain a high-fidelity printed product, preliminary experiments were conducted to determine the optimal printing parameters. The optimal printing parameters were noted as: nozzle diameter of 1.2 mm, layer height of 6 mm, infill percentage of 60, printing speed of 70 mm/s, extrusion rate of 79.17 mm^3^/s, extrusion temperature of 25 °C and travel speed of 130 mm/s. After printing, 3D-printed meat mimics were incubated in a refrigerator at 4 °C for 24 h. Each incubated sample was left at room temperature (25 ± 1 °C) for 30 min prior to cooking and/or texture analysis.

### 2.6. Cooking of 3D-Printed Meat Mimics

3D-printed meat mimics were cooked via pan frying. The cooking process started by heating a Teflon pan with an electric stove (Tefal, IH201868, Bangkok, Thailand) using a frying program (C5) until the temperature of the pan reached 150 °C. Around 5 mL of soybean oil (Morakot, Samutprakarn, Thailand) was poured into the pan and heated for 0.5 min. A sample was then placed in the pan and heated until its core temperature reached 80 °C. The total cooking time was 10 min (3 min for the top and bottom sides and 2 min for the left and right sides). Temperatures of the pan and meat mimics were continuously measured using a digital thermocouple (Yokogawa, TX10, Tokyo, Japan). After cooking, the fried sample was left at room temperature (25 ± 1 °C) for 30 min prior to texture analysis.

To determine the cooking loss, mass of uncooked meat mimics (raw) and that of the cooked one were measured. Cooking loss was calculated using Equation (1).

(1) $$\%\;\mathrm{Cooking loss}=\frac{W_{1}-W_{2}}{W_{1}}\times 100$$

where *W*_1_ is the mass of raw meat mimics (g), while *W*_2_ is the mass of cooked meat mimics (g).

### 2.7. Textural Properties Determination of 3D-Printed Meat Mimics

**Figure 2:**
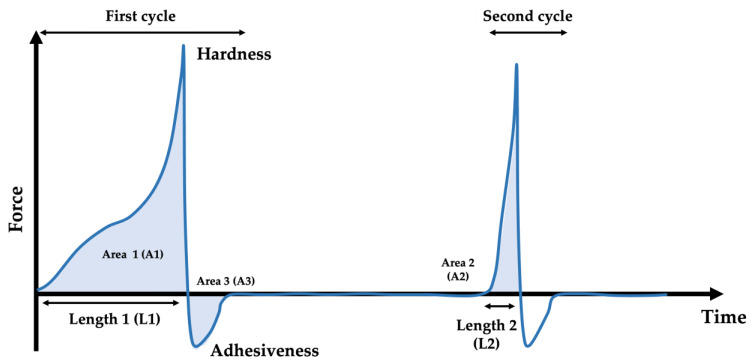
A typical force–time curve.

Textural properties of raw and cooked meat mimics were determined using a texture analyzer (Stable Micro Systems, TA.XTplus, Surrey, UK) as per the modified methods of Ruiz de Huidobro et al. [23]. A sample was cut into a 2 × 2 cm strip. A 50-mm diameter cylindrical probe was used for the determination. The probe was allowed to move downward at a pre-test speed of 1.0 mm/s, test speed of 1.0 mm/s and post-test speed of 3.0 mm/s. The probe continued downward to 75% strain, returned to the initial point of contact with the sample and stopped for a pre-determined period of time (2 s) before the second compression cycle. During each cycle, the resistance of the sample was recorded at every 0.01 s and plotted into a force–time curve (Figure 2). Textural properties are reported in terms of hardness, chewiness, springiness, adhesiveness and cohesiveness.

Textural properties were calculated from the force–time curve as per Equations (2)–(4) [24].

Chewiness = A2/A1 × Hardness × Springiness(2)

Springiness = L2/L1(3)

Cohesiveness = A2/A1(4)

The following definitions of textural properties were adopted: hardness is the force required to deform a sample to a given distance; chewiness is the energy required to chew a sample until it is ready for swallowing; springiness is the rate at which a deformed sample goes back to its non-deformed condition once the deforming force has been removed; cohesiveness is the intermolecular attractive force acting between two adjacent portions of a sample; and adhesiveness is the force required to remove a sample that adheres to the probe surface.

### 2.8. Comparison with Animal Meat

Textural properties of raw and cooked meat mimics were compared with those of selected raw and cooked animal meats, namely, pork tenderloin, chicken breast, salmon and Spanish mackerel. Both raw and cooked animal meat was analyzed by using the aforementioned protocols. In the case of raw animal meat, a sample was kept at room temperature for 30 min prior to texture analysis. In the case of cooked animal meat, cuts of the raw meat (with the dimensions of 20 × 80 × 25.4 mm) were cooked at the same conditions used for 3D-printed samples.

### 2.9. Statistical Analysis

The experimental data were subject to the analysis of variance (ANOVA) and are presented as mean values with standard deviations. Tukey’s test was used to establish significance of the differences among the mean values at a confidence level of 95%. All statistical calculations were performed using Minitab version 19.0 (Minitab, State College, PA, USA). All experiments were performed in triplicate.

## 3. Results and Discussion

### 3.1. Rheological Properties of Printing Material

Relevant viscoelasticity values of a 3D-printing food material should be high enough to provide shape retention after printing, but low enough to allow extrusion through a small nozzle during the printing process [25]. Self-supporting ability, extrudability and printability of a material depend on its storage modulus (*G′*), loss modulus (*G″*) and tan δ (ratio of *G″* to *G′*). A low tan δ value is beneficial for maintaining the structure of a product after printing [26,27]. Nevertheless, a tan *δ* value that is too low (lower than 0.1) implies that a material would be difficult to extrude, resulting in broken lines, i.e., poor print quality [28].

**Figure 3:**
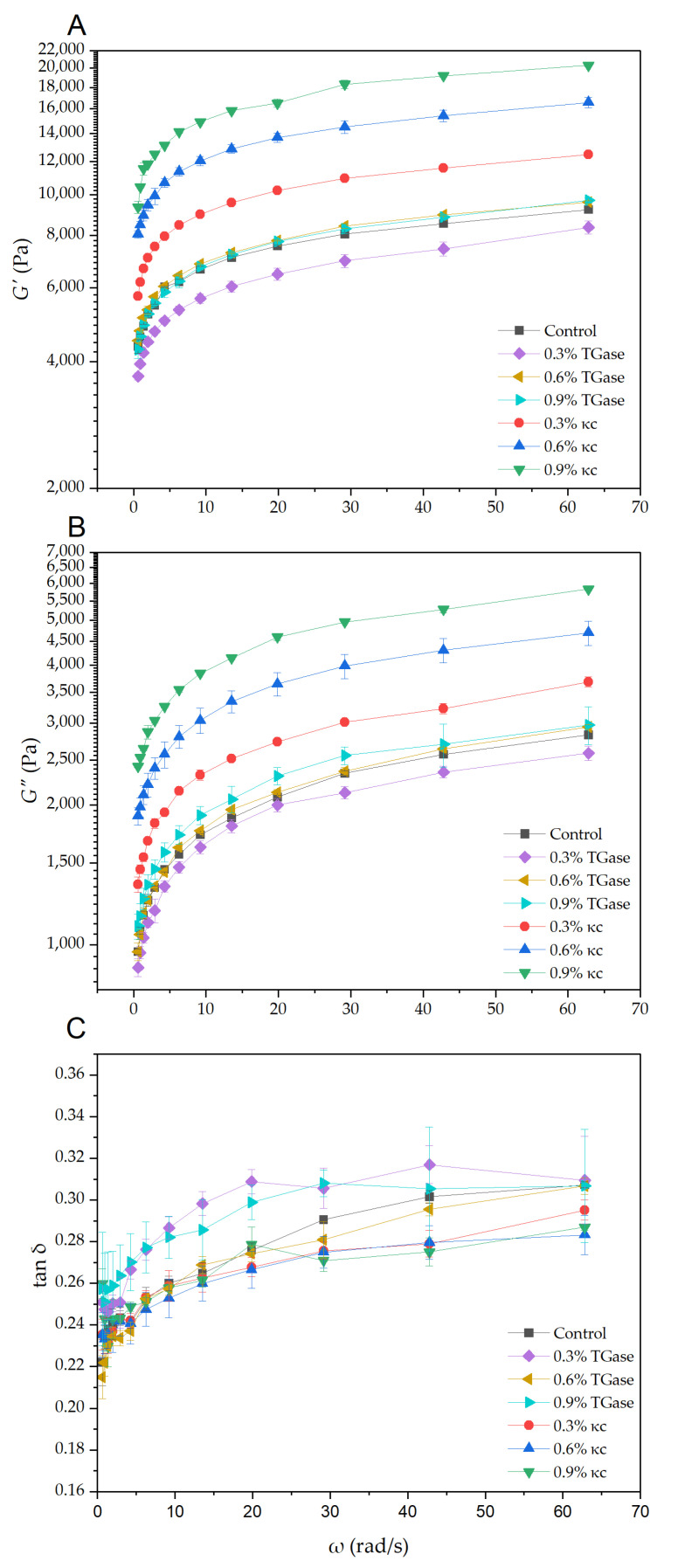
(**A**) Storage modulus (*G′*); (**B**) loss modulus (*G″*); and (**C**) loss tangent (tan δ = *G″*/*G′*) vs. angular frequency of printing material treated with TGase and κc at 0.3, 0.6 and 0.9%.

*G′*, *G″* and tan δ values of the printing material are shown in Figure 3. In all cases, *G′* values were higher than *G″* values. This indicates that the material exhibited the ability to form an elastic gel or a gel-like structure. In the case of TGase-modified material, *G′*, *G″* and tan δ values were not significantly different when compared with those of the control. This is due to the fact that the rheological properties measurement was made prior to the printing; no incubation or heat treatment of the modified printing material was performed. TGase might not have yet cross-linked the protein molecules since enzyme activity is both temperature- and time-dependent [29]. The tan δ values of the samples modified with TGase at 0.3%, 0.6% and 0.9% were also not significantly different, being in the ranges of 0.25–0.31, 0.21–0.31 and 0.26–0.31, respectively. The values of the control were in the range of 0.22–0.31.

In the case of κc-modified material, increasing the κc content resulted in an increase in *G′* and *G″*. The tan δ values of all the κc-modified samples decreased from that of the control and were 0.23–0.29, 0.23–0.28 and 0.26–0.29 at the κc contents of 0.3%, 0.6% and 0.9%, respectively. This is probably because of the change in the polysaccharides–protein complex, resulting in the change in the rheological properties [30]. Decrease in the loss tangent value indicates a decrease in the fluidity of the material, resulting in turn in its lower extrudability and printability [28,31].

### 3.2. Appearances of 3D-Printed Meat Mimics

**Figure 4:**
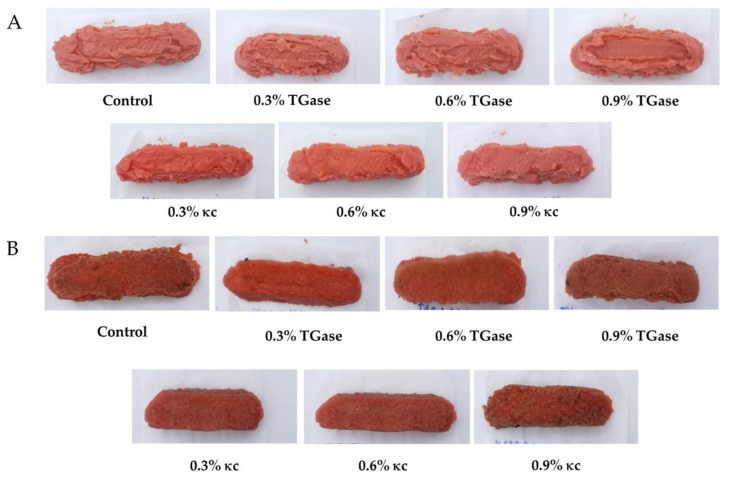
Appearances of (**A**) raw and (**B**) cooked meat mimics treated with TGase and κc at 0, 0.3, 0.6 and 0.9%.

The appearances of both raw and cooked meat mimics treated with TGase and κc are shown in Figure 4. In the case of the raw meat mimics, the appearances of all the printed samples were similar. However, based on an internal visualization of the structure, meat mimics treated with κc appeared more relaxed with more pores. This is probably due to the aforementioned lower tan δ values, leading to the reduced printability (i.e., adhesion between layers) and more pores within the printed structure. Such relaxed structure resulted in the different hardness values of the meat mimics, as will be discussed in Section 3.3.1.

In the case of the cooked meat mimics, although all the cooked samples possessed similar appearances, their textural properties were significantly different. The results will be discussed in Section 3.3.2. Cooked meat mimics exhibited a darker color than raw meat mimics. This is expected as the Maillard reaction took place during the cooking [32].

### 3.3. Textural Properties of 3D-Printed Meat Mimics

#### 3.3.1. Raw Meat Mimics

Textural properties, in terms of hardness, chewiness, springiness, adhesiveness and cohesiveness, of the raw meat mimics are listed in Table 1. Increasing the TGase content resulted in an increase in the hardness of the raw meat mimics, due most probably to the formation of crosslinked isopeptide bonds between lysine and glutamine residues [33]. The hardness of TGase-treated meat mimics became closer to that of animal meat. In the case of the κc-treated meat mimics, the hardness of the samples decreased; this is related to the decrease in the printability of the κc-treated printing material. The decreased printability adversely affected the continuity of the printing material depositions. Such lack of continuity resulted in the aforementioned relaxed structure of the printed meat mimics and hence the decreased hardness when compared with that of the control.

All treated meat mimics possessed lower chewiness, springiness (or elasticity), adhesiveness and cohesiveness than the control. The decrease in the cohesiveness led to the decrease in the chewiness. This is because the treated samples were more like a brittle gel [34]; the decrease in the springiness was related to the decrease in the elasticity of the treated samples. TGase-treated meat mimics exhibited higher chewiness, springiness and adhesiveness than κc-treated ones, probably because of the lower porosity of the former. However, further analysis via such techniques as micro-computed tomography is needed to verify this hypothesis.

#### 3.3.2. Cooked Meat Mimics

Although the raw meat mimics treated with TGase exhibited higher hardness values than the control, the hardness values of the cooked meat mimics treated with TGase as well as those of the cooked control were not significantly different (Table 2). This is probably because of the loss in the TGase activity upon cooking. Some covalent bonds that were formed might have also been broken during the high-temperature pan frying process. In the case of the cooked κc-treated meat mimics, the hardness of the samples did not significantly differ from that of the control, except in the case of the sample with κc at 0.9%. This might be related to the higher loss of moisture of the meat mimics treated with 0.9% κc upon cooking. Rigidity of the sample therefore increased, leading to the increased hardness [35].

Chewiness values of all the cooked samples were similar to that of the raw ones, except in the case of the sample treated with 0.9% κc. This is again because using κc at a higher concentration caused a decrease in the moisture content of the sample when its temperature reached around 70 °C [35]. This led in turn to the higher required energy to chew the sample and hence the increased chewiness value. The increase in the chewiness resulted in the texture of the meat mimics being more similar to that of animal meat.

Loss of moisture upon cooking caused a decrease in the springiness, adhesiveness and cohesiveness. Upon cooking, the sample treated with 0.9% κc, which suffered the highest moisture loss, possessed the highest hardness value [36]. Moisture loss of each sample upon cooking was determined in terms of cooking loss, which will be discussed in the next section.

### 3.4. Cooking Losses of 3D-Printed Meat Mimics

**Figure 5:**
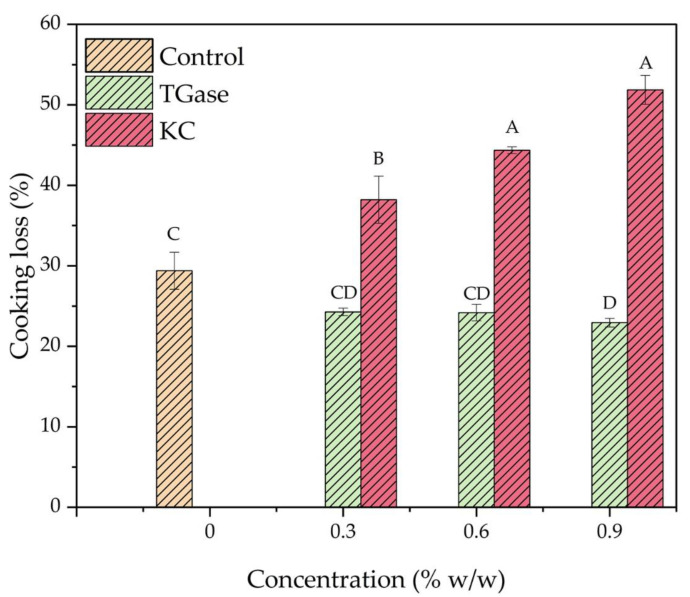
Effect of texture modification with TGase and κc at 0, 0.3, 0.6 and 0.9% on cooking loss of meat mimics. Different letters over bars imply that the values are significantly different (*p* < 0.05).

Cooking losses of meat mimics treated with TGase and κc are shown in Figure 5. Addition of TGase tended to help decrease the loss of moisture, while the samples treated with κc experienced the higher loss of moisture than the control. Increasing the κc content resulted in an increase in the cooking loss, as mentioned earlier. These observations are ascribed to the fact that TGase resulted in the higher ability to retain water within the printed meat mimics as a result of enzymatic crosslinking. κc, on the other hand, did not exhibit such a capability. Cooking losses of the control, samples treated with TGase at 0.3%, 0.6%, 0.9% and samples treated with κc at 0.3%, 0.6% and 0.9% were 29.39, 24.27, 24.18, 22.93, 38.21, 44.36 and 51.85%, respectively.

### 3.5. Comparison between 3D-Printed Meat Mimics and Animal Meats

To preliminarily identify which real animal meat the 3D-printed meat mimics of the present study could resemble, the textural characteristics of several animal meats were determined. Raw and cooked pork tenderloin, chicken breast, salmon and Spanish mackerel were chosen as the test animal meats; their textural properties are listed in Table 1 and Table 2. The results revealed that the hardness, chewiness and springiness of raw meat mimics treated with TGase at 0.9% were closest to those of salmon meat, while the cohesiveness was closer to that of pork tenderloin. However, adhesiveness of the raw meat mimics was higher than that of all animal meats. For cooked meat mimics, the hardness, chewiness, springiness and adhesiveness of the meat mimics treated with κc at 0.9% were closer to those of cooked salmon meat.

## 4. Conclusions

3D printing was used to prepare plant-based meat mimics from a mixture of pea protein isolate and alginate gel. Texture modifiers (TGase and κc) at different concentrations were used to modify the printing material prior to 3D printing. The tan δ values of TGase-modified printing material were not significantly different from that of the control, while the values belonging to all κc-modified material were lower than that of the control. All printed samples externally appeared similarly. Cooked meat mimics were nevertheless expectedly darker than raw meat mimics. Hardness of raw meat mimics treated with 0.9% (*w/w*) TGase was higher, while the values belonging to raw meat mimics treated with κc at all concentrations were lower than that of the control, due probably to the more relaxed structure of the latter. In the case of cooked meat mimics, hardness values of the samples treated with TGase were not significantly different from that of the control, while cooked 0.9% κc-treated meat mimics exhibited the highest hardness of 37.14 N. This might be related to the more extensive loss of moisture upon cooking; rigidity of the sample therefore increased, leading to the increased hardness. When compared with selected animal meats, textural properties of raw meat mimics treated with TGase at 0.9% (*w/w*) were closest to those of raw salmon. Textural properties of cooked meat mimics treated with κc at 0.9% (*w/w*) were also closest to those of cooked salmon. Further development to improve the texture of 3D-printed meat mimics is clearly needed. This can probably be accomplished by incorporating fat layers or fibrous structure into the printed matrix via smart infill designs.

## Figures and Tables

**Table 1:** Textural properties of raw 3D-printed meat mimics treated by TGase and κc at 0, 0.3, 0.6 and 0.9% as well as those of raw animal meats.

Sample	Hardness (N)	Chewiness (N)	Springiness	Adhesiveness (N·s)	Cohesiveness
Control	7.30 ± 1.19 ^e^	173.08 ± 20.01 ^c^	0.49 ± 0.04 ^a^	−17.22 ± 4.11 ^d^	0.47 ± 0.02 ^a^
0.3% TGase	8.10 ± 1.01 ^e^	57.38 ± 17.67 ^c^	0.24 ± 0.05 ^b^	−9.64 ± 1.71 ^c^	0.29 ± 0.03 ^b^
0.6% TGase	8.74 ± 0.61 ^de^	51.34 ± 8.71 ^c^	0.23 ± 0.03 ^b^	−7.84 ± 3.05 ^bc^	0.25 ± 0.03 ^bc^
0.9% TGase	10.48 ± 1.09 ^de^	61.27 ± 7.26 ^c^	0.25 ± 0.20 ^b^	−6.20 ± 2.02 ^bc^	0.23 ± 0.04 ^bc^
0.3% κc	6.23 ± 0.66 ^e^	41.96 ± 5.38 ^c^	0.24 ± 0.02 ^b^	−6.67 ± 1.01 ^bc^	0.28 ± 0.01 ^b^
0.6% κc	5.51 ± 0.56 ^e^	31.75 ± 8.67 ^c^	0.21 ± 0.03 ^b^	−5.71 ± 0.42 ^bc^	0.27 ± 0.04 ^b^
0.9% κc	5.76 ± 0.53 ^e^	27.83 ± 14.79 ^c^	0.19 ± 0.07 ^b^	−4.43 ± 0.72 ^ab^	0.24 ± 0.04 ^bc^
Pork tenderloin	149.89 ± 11.78 ^a^	1573.03 ± 275.68 ^a^	0.48 ± 0.13 ^b^	−0.67 ± 0.16 ^a^	0.25 ± 0.09 ^b^
Chicken breast	79.06 ± 8.41 ^b^	515.65 ± 135.69 ^b^	0.34 ± 0.04 ^b^	−0.91 ± 0.25 ^a^	0.17 ± 0.03 ^cd^
Salmon	16.45 ± 1.81 ^d^	49.82 ± 11.41 ^c^	0.26 ± 0.05 ^b^	−0.97 ± 0.73 ^a^	0.11 ± 0.01 ^d^
Spanish mackerel	25.38 ± 2.19 ^c^	109.97 ± 32.84 ^c^	0.25 ± 0.08 ^b^	−3.76 ± 0.65 ^ab^	0.15 ± 0.02 ^d^

Different letters in the same column indicate that the values are significantly different (*p* < 0.05).

**Table 2:** Textural properties of cooked 3D-printed meat mimics treated by TGase and κc at 0, 0.3, 0.6 and 0.9% as well as those of cooked animal meats.

Sample	Hardness (N)	Chewiness (N)	Springiness	Adhesiveness (N·s)	Cohesiveness
Control	20.07 ± 0.95 ^e^	150.04 ± 31.53 ^d^	0.34 ± 0.07 ^a^	−2.65 ± 0.87 ^cd^	0.22 ± 0.02 ^d^
0.3% TGase	11.32 ± 2.48 ^e^	59.32 ± 19.50 ^d^	0.20 ± 0.04 ^b^	−2.04 ± 0.99 ^bcd^	0.21 ± 0.02 ^d^
0.6% TGase	15.59 ± 2.50 ^e^	63.68 ± 18.34 ^d^	0.21 ± 0.03 ^ab^	−1.90 ± 0.50 ^bcd^	0.21 ± 0.02 ^d^
0.9% TGase	19.11 ± 1.36 ^e^	87.58 ± 10.86 ^d^	0.26 ± 0.02 ^ab^	−1.83 ± 0.93 ^bcd^	0.17 ± 0.01 ^d^
0.3% κc	19.48 ± 0.78 ^e^	68.17 ± 12.01 ^d^	0.19 ± 0.03 ^b^	−1.21 ± 0.41 ^abc^	0.18 ± 0.02 ^d^
0.6% κc	16.44 ± 1.28 ^e^	57.31 ± 12.18 ^d^	0.18 ± 0.01 ^b^	−1.55 ± 0.85 ^bc^	0.19 ± 0.05 ^d^
0.9% κc	37.14 ± 3.85 ^de^	156.16 ± 18.29 ^d^	0.21 ± 0.05 ^ab^	−1.26 ± 0.13 ^abc^	0.21 ± 0.04 ^d^
Pork tenderloin	246.42 ± 30.89 ^a^	6510.83 ± 1509.93 ^a^	0.49 ± 0.07 ^ab^	−1.59 ± 0.89 ^bc^	0.51 ± 0.02 ^a^
Chicken breast	175.71 ± 10.18 ^b^	2360.66 ± 385.27 ^c^	0.41 ± 0.06 ^ab^	−3.15 ± 0.91 ^d^	0.29 ± 0.02 ^c^
Salmon	47.55 ± 10.84 ^d^	932.36 ± 567.65 ^cd^	0.42 ± 0.10 ^ab^	−0.88 ± 0.33 ^ab^	0.41 ± 0.06 ^b^
Spanish mackerel	149.02 ± 2.70 ^c^	4241.37 ± 727.12 ^b^	0.25 ± 0.08 ^ab^	−0.02 ± 0.02 ^a^	0.49 ± 0.03 ^ab^

Different letters in the same column indicate that values are significantly different (*p* < 0.05).

## Data Availability

Experimental and relevant data are available from the authors upon suitable request.

## References

[1] Steinfeld H., Gerber P., Wassenaar T.D., Castel V., Rosales M., de Haan C. (2006). Livestock’s Long Shadow: Environmental Issues and Options.

[2] Vinnari M., Tapio P. (2009). Future images of meat consumption in 2030. Futures.

[3] Elzerman J.E., Hoek A.C., van Boekel M.A.J.S., Luning P.A. (2011). Consumer acceptance and appropriateness of meat substitutes in a meal context. Food Qual Prefer..

[4] Hoek A.C., Luning P.A., Weijzen P., Engels W., Kok F.J., de Graaf C. (2011). Replacement of meat by meat substitutes. A survey on person- and product-related factors in consumer acceptance. Appetite.

[5] Michel F., Hartmann C., Siegrist M. (2021). Consumers’ associations, perceptions and acceptance of meat and plant-based meat alternatives. Food Qual. Prefer..

[6] Dekkers B.L., Boom R.M., van der Goot A.J. (2018). Structuring processes for meat analogues. Trends Food Sci. Technol..

[7] Phuhongsung P., Zhang M., Bhandari B. (2020). 4D printing of products based on soy protein isolate via microwave heating for flavor development. Food Res. Int..

[8] Phuhongsung P., Zhang M., Devahastin S. (2020). Investigation on 3D printing ability of soybean protein isolate gels and correlations with their rheological and textural properties via LF-NMR spectroscopic characteristics. LWT-Food Sci. Technol..

[9] Wang S., Lui S. (2021). 3D printing of soy protein- and gluten-based gels facilitated by thermosensitive cocoa butter in a model study. ACS Food Sci. Technol..

[10] Mackay M.E. (2018). The importance of rheological behavior in the additive manufacturing technique material extrusion. J. Rheol..

[11] Oyinloye T.M., Yoon W.B. (2021). Stability of 3D printing using a mixture of pea protein and alginate: Precision and application of additive layer manufacturing simulation approach for stress distribution. J. Food Eng..

[12] Zhao H., Shen C., Wu Z., Zhang Z., Xu C. (2020). Comparison of wheat, soybean, rice, and pea protein properties for effective applications in food products. J. Food Biochem..

[13] Barać M., Cabrilo S., Pešić M., Stanojević S., Pavlićević M., Maćej O., Ristić N. (2011). Functional properties of pea (*Pisum sativum,* L.) protein isolates modified with chymosin. Int. J. Mol. Sci..

[14] Kim H.W., Lee I.J., Park S.M., Lee J.H., Nguyen M.-H., Park H.J. (2019). Effect of hydrocolloid addition on dimensional stability in post-processing of 3D printable cookie dough. LWT-Food Sci. Technol..

[15] Carvajal-Piñero J.M., Ramos M., Jiménez-Rosado M., Perez-Puyana V., Romero A. (2019). Development of pea protein bioplastics by a thermomoulding process: Effect of the mixing stage. J. Polym. Environ..

[16] Kim H.S., Lee C.-G., Lee E.Y. (2011). Alginate lyase: Structure, property and application. Biotechnol. Bioprocess Eng..

[17] Wang J., Wei J., Su S., Qiu J., Wang S. (2015). Ion-linked double-network hydrogel with high toughness and stiffness. J. Mater. Sci..

[18] Tsai. C.-R., Lin Y.-K. (2022). Artificial steak: A 3D printable hydrogel composed of egg albumen, pea protein, gellan gum, sodium alginate and rice mill by-products. Future Foods.

[19] Swidsinski A., Ung V., Sydora B.C., Loening-Baucke V., Doerffel Y., Verstraelen H., Fedorak R.N. (2009). Bacterial overgrowth and inflammation of small intestine after carboxymethylcellulose ingestion in genetically susceptible mice. Inflamm. Bowel Dis..

[20] Sarteshnizi R., Hosseini H., Mousavi Khaneghah A., Karimi N. (2015). A review on application of hydrocolloids in meat and poultry products. Int. Food Res. J..

[21] Setiadi S.W.I., Alisha N. (2018). The influences of transglutaminase enzyme dosage on the meat characteristic from restructuring the animal and vegetable protein sources. E3S Web Conf. 3rd Int. Trop. Renew. Energy Conf..

[22] Tunnarut D., Nopwinyuwong A., Tanakamolpradit T. (2022). Quality improvement of plant-based patty using methylcellulose, κ-carrageenan and xanthan gum. JCST.

[23] Ruiz de Huidobro F., Miguel E., Blázquez B., Onega E. (2005). A comparison between two methods (Warner-Bratzler and texture profile analysis) for testing either raw meat or cooked meat. Meat Sci..

[24] Szczesniak A. (1963). Classification of textural characteristics. J. Food Sci..

[25] Godoi F.C., Prakash S., Bhandari B.R. (2016). 3D printing technologies applied for food design: Status and prospects. J. Food Eng..

[26] Liu Y., Liang X., Saeed A., Lan W., Qin W. (2019). 2019. Properties of 3D printed dough and optimization of printing parameters. Innov. Food Sci. Emerg. Technol..

[27] Guo C., Zhang M., Devahastin S. (2020). 3D extrusion-based printability evaluation of selected cereal grains by computational fluid dynamic simulation. J. Food Eng..

[28] Liu Z., Zhang M., Bhandari B., Wang Y. (2017). 3D printing: Printing precision and application in food sector. Trends Food Sci. Technol..

[29] Rossa P.N., de Sá E.M.F., Burin V.M., Bordignon-Luiz M.T. (2011). Optimization of microbial transglutaminase activity in ice cream using response surface methodology. LWT-Food Sci. Technol..

[30] Ghosh A.K., Bandyopadhyay P., Karunaratne D.N. (2012). Polysaccharide-protein interactions and their relevance in food colloids. The Complex World of Polysaccharides.

[31] Yang F., Zhang M., Bhandari B., Liu Y. (2018). Investigation on lemon juice gel as food material for 3D printing and optimization of printing parameters. LWT-Food Sci. Technol..

[32] Shahidi F., Samaranayaka A.G.P., Pegg R.B., Dikeman M., Devine C. (2014). Maillard reaction and browning. Encyclopedia of Meat Sciences.

[33] Hiller B., Lorenzen P.C. (2009). Effect of phosphatase/transglutaminase treatment on molar mass distribution and techno-functional properties of sodium caseinate. LWT-Food Sci. Technol..

[34] Pietrasik Z. (2003). Binding and textural properties of beef gels processed with κ-carrageenan, egg albumin and microbial transglutaminase. Meat Sci..

[35] Foegeding E.A., Ramsey S.R. (1987). Rheological and water-holding properties of gelled meat batters containing iota carrageenan, kappa carrageenan or xanthan gum. J. Food Sci..

[36] Vu G., Zhou H., McClements D.J. (2022). Impact of cooking method on properties of beef and plant-based burgers: Appearance, texture, thermal properties and shrinkage. J. Agric. Food Res..

